# Rheological and Aging Properties of Vegetable Oil-Based Polyurethane (V-PU) Modified Asphalt

**DOI:** 10.3390/polym15092158

**Published:** 2023-04-30

**Authors:** Lei Xia, Dongwei Cao, Hongliang Zhang

**Affiliations:** 1 School of Materials Science and Engineering, Chang’an University, Xi’an 710064, China; 2 China-Road Transportation Verification & Inspection Hi-Tech Co., Ltd., RIOH, Beijing 100088, China; 3 Key Laboratory for Special Area Highway Engineering of Ministry of Education, Chang’an University, Xi’an 710064, China

**Keywords:** polyurethane, vegetable oil, modified asphalt, rheological properties, aging properties, aging mechanism

## Abstract

To study the rheological and aging properties of vegetable oil–based polyurethane (V-PU) modified asphalt, V-PU terminated with an –NCO group was synthesized from renewable castor oil, and liquefied MDI-100LL and 10–40 wt% V-PU modified asphalts were prepared. Temperature classification, multiple stress creep recovery (MSCR), and linear amplitude scanning (LAS) tests were carried out. The results showed that the modulus, the creep recovery rate (R), and the yield stress and yield strain of the V-PU modified asphalts significantly increased in the order: 0 wt% < 10 wt% < 20 wt% < 40 wt% < 30 wt%, while the phase angle and the unrecoverable creep compliance (Jnr) changed in the opposite order, and the high temperature grade of 30 wt% V-PU modified asphalt was 4 grades higher than that of the base asphalt, which indicated that the addition of V-PU enhanced the fatigue, permanent deformation, and recovery deformation resistance. The 30 wt% sample exhibited phase inversion had the best performance. Comprehensive FTIR, GPC, and fluorescence microscopy analyses showed that the molecular weight significantly increased and the V-PU molecules agglomerated after aging. The excess –NCO groups of V-PU prepolymer react with water in the air and the active hydrogen in the asphalt system and finally form a cross-linked three-dimensional network structure with the asphalt to improve performance. The mechanism of intramolecular cementation reaction and the aging process of V-PU modified asphalt was creatively derived.

## 1. Introduction

Asphalt is a road material with rheological properties under the general service conditions of −30–70 °C. The flow and deformation are related to stress, temperature, and time. The rheological property of asphalt is a key factor affecting the use of asphalt pavement. Asphalt shows both viscous and elastic properties in the use temperature range. To improve the viscoelastic behavior of asphalt binders, engineers and material scientists have made attempts to modify the properties of asphalt binders and mixtures using different types of modifiers [1,2,3]. An ideal modifier improves the properties of an asphalt binder by increasing its modulus at high temperatures and reducing its brittleness at low temperatures [4].

In recent years, various polymers such as styrene butadiene styrene (SBS), styrene butadiene rubber (SBR), ethylene vinyl acetate (EVA), high-density polyethylene (HDPE), low-density polyethylene (LDPE), resins, lignin, and rubber have received significant attention for enhancing the performance of asphalt binders and mixtures [5,6,7,8,9,10,11,12,13,14,15,16,17,18,19]. The addition of polymers to asphalt increases its stiffness and viscosity and reduces its temperature susceptibility [20]. In addition, polymer modified asphalt mixtures have been found to show improved rutting resistance and fatigue performance [21].

**Scheme 1:**
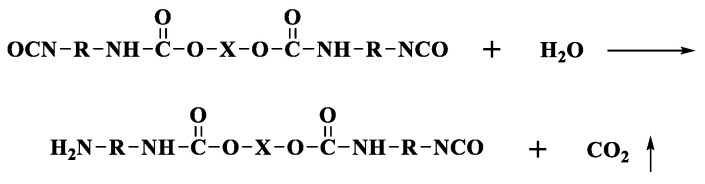
(–NHCOO–) group in the PU molecular structure reacts with active hydrogen groups in the asphalt system.

Polyurethane (PU) is a novel asphalt modifier that has a special combination structure of soft and hard segments with different crystallinities and which are thermodynamically incompatible with each other, similarly to SBS [22]. The (–NHCOO–) group in the PU molecular structure reacts with active hydrogen groups in the asphalt system, which can improve the resistance to permanent deformation (Scheme 1) [23]. Li reported optimization of the PU modified asphalt preparation process by observing the curing rate and mixing uniformity of PU. It was found that the modification effect was optimal when the stirring temperature was 120 °C and the curing time was 48 h [24]. Liu determined the best preparation parameters for PU modified asphalt using an orthogonal test [25]. Bazmara used thermoplastic PU to modify the asphalt and showed that the penetration of modified asphalt decreased, the softening point increased, and the high-temperature deformation resistance greatly improved [26]. Zhang prepared a new type of PU thermoplastic elastomer as an asphalt modifier and found that PU elastomer modified asphalt had better low-temperature crack resistance and water stability than SBS modified asphalt [27]. Yan prepared bone glue/PU composite modified asphalt (CMA), determined the optimal dosage with 5–10% bone glue and 1–5% PU, and revealed the relationship between the modifier’s content and the conventional properties and rheological properties of CMA [28]. Chen replaced part of the SBS modifier with PU for preparing composite modified asphalt. He found that SBS/PU composite modified asphalt had good low-temperature resistance and that its high-temperature performance was lower than that of SBS modified asphalt; He determined 4% SBS content and 15% PU content as the suitable types of SBS/PU composite modified asphalt [29]. The PU/epoxy resin underwent a reaction with the curing agent to form an interpenetrating polymer network structure in a PU/epoxy resin composite modified asphalt system and exhibited excellent mechanical properties and thermal storage stability [30,31]. Yu analyzed the weakening of asphalt characteristic peaks in the FTIR spectrum and reported that the isocyanate group reacted with active hydrogen in the asphalt to form cross-linking, and the PU chain was broken and degraded after aging [32]. Sun found that two chemical reactions occurred: the reaction between isocyanate and polyol to generate carbamate, and the additional reaction between isocyanate and aromatic compounds in asphaltene after PU was added to the base asphalt [33]. 

However, most previous studies have focused on optimization of the preparation process of PU modified asphalt, the performance of SBS/PU and PU/epoxy composite modified asphalt, and the preliminary exploration of the possible reaction mechanism of PU modified asphalt. Unfortunately, both the process and the additives were complex, adding to the cost, and the high performance was mostly dependent on the high strength of epoxy or traditional SBS modifier. Vegetable oil is sustainable, environmentally friendly, and naturally renewable, with a low price. Moreover, castor oil has high hydroxyl value and can react with isocyanate as polyol at room temperature with relatively low energy consumption [34]. In addition, there have been few studies of the rheological and mechanical properties of PU modified asphalt, particularly the fatigue properties. Exploring the intramolecular bonding reaction mechanism and the aging process of V-PU modified asphalt would be innovative.

In our previous study, vegetable oil–based polyurethane (V-PU) was synthesized with recycled vegetable oil instead of polyol and liquefied MDI-100LL at room temperature, and 10–40 wt% V-PU modified asphalts were prepared. The strength, permanent deformation resistance, and fatigue cracking resistance were compared and evaluated on a dynamic shear rheometer (DSR). The strength formation mechanism and the structure change process of PU modified asphalt during aging were analyzed.

## 2. Materials and Methods

### 2.1. Materials

**Figure 1:**
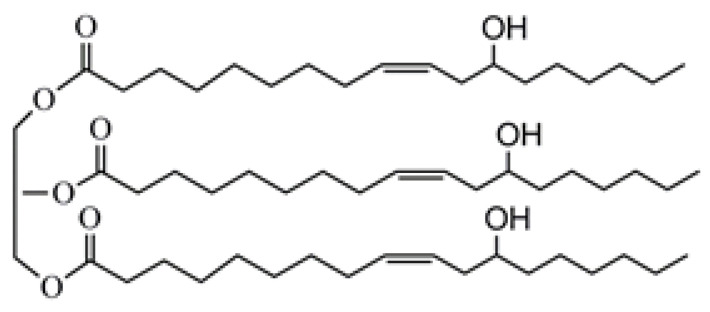
Molecular structure of castor oil.

**Figure 2:**
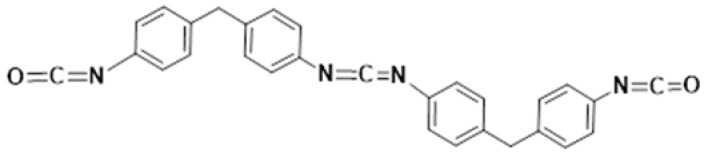
Molecular structure of liquefied MDI-100LL.

Vegetable oil–based polyol: the castor oil (AR) was provided by Sinopharm Group Co., Ltd. (Beijing, China). The molecular structure is shown in Figure 1, and the base properties are given in Table 1. The liquefied MDI-100LL was supplied by Wanhua Chemical Industry Group Co., Ltd. (Beijing, China). The molecular structure is shown in Figure 2, and the base properties in Table 2. The dibutyl dilaurate (AR) catalyst, phosphoric acid (AR) polymerization inhibitor, and acetone (AR) were obtained from Beijing Chemical Factory Co., Ltd. (Beijing, China). The base asphalt was 70# road petroleum asphalt, and the base properties are shown in Table 3, with technical requirements on the basis of JTG F40-2004, Technical Specifications for Construction of Highway Asphalt Pavements [35].

The amount of castor oil and liquefied MDI-100LL and the isocyanate index (R) were calculated as follows:

(1) $$R=\frac{n_{-\mathrm{NCO}}}{n_{-\mathrm{OH}}}=\frac{m_{\mathrm{MDI}}\times w_{-\mathrm{NCO}}\%\times 17}{42\times m_{\mathrm{Co}}\times w_{-\mathrm{OH}}\%},$$

(2) $$m_{\mathrm{MDI}}=\frac{m_{\mathrm{Co}\times R\times 42\times w_{–\mathrm{OH}}\%}}{17\times w_{–\mathrm{NCO}}\%}$$

where R is isocyanate index, with a value of 1.2, m_MDI_ is the mass of liquefied MDI-100LL, w_–NCO_% is –NCO content, m_Co_ is the mass of castor oil, and w_–OH_% is –OH content.

### 2.2. Preparation of V-PU

The vegetable oil was vacuum dehydrated at 110 °C, 0.1 MPa in a vacuum drying box for 2–3 h and then cooled. Phosphoric acid, which was used as a polymerization inhibitor, was added to a dry four-necked flask. Then the liquefied MDI-100LL was added and the mixture was stirred for several minutes. The required mass of dehydrated vegetable oil was calculated according to Equations (1) and (2) and dropped into the four-necked flask dropwise at room temperature under an atmosphere of nitrogen. The dropping speed was controlled and the viscosity and rate of reaction were adjusted with acetone. The reaction was allowed to proceed for several minutes after the vegetable oil was added to gain V-PU terminated with –NCO groups. The –NCO content was determined, and the sample was degassed and discharged for storage.

### 2.3. Preparation of V-PU Modified Asphalt

The V-PU modified asphalt was prepared in two steps. First, 70# base asphalt was heated to 140 °C in an oven for 3 h. Then, V-PU was added to the base asphalt with the process conditions: sheared at least 50 min at 120–130 °C with a Reynolds number of about 845–1500 to obtain a homogeneous phase.

**Figure 3:**
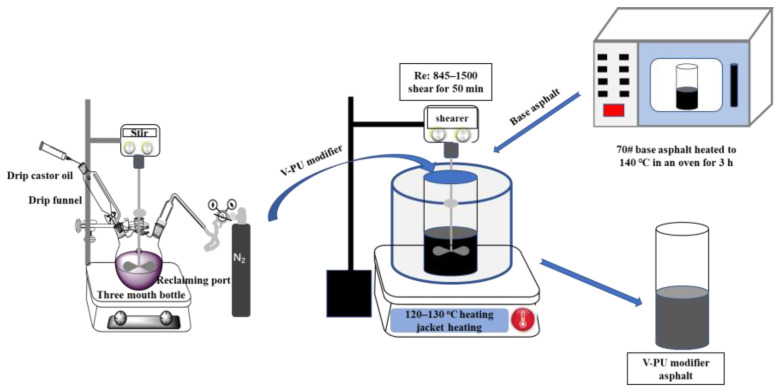
Schematic diagram of V-PU synthesis and V-PU modified asphalt preparation method.

A schematic diagram of V-PU synthesis and the V-PU modified asphalt preparation method is shown in Figure 3.

### 2.4. Classical Performance

Classical performance testing included penetration, softening point, ductility, the thin-film oven test (TFOT), and the pressure aging vessel accelerated asphalt aging test (PAV) according to JTG E 20-2011, Standard Test Methods of Bitumen and Bituminous Mixtures for Highway Engineering [36].

### 2.5. Rheological Properties

The dynamic rheological properties of modified asphalt were tested using a dynamic shear rheometer (DSR) (Anton Paar, Graz, Austria). High-temperature classification tests, MSCR, and LAS tests were conducted to compare the mechanical properties of the V-PU modified asphalt. The specific methods were as follows:

(1) High temperature classification test

The DSR was used to conduct vibration scanning on the original base asphalt, the V-PU modified asphalt, and the TFOT residual asphalt. Conditions: Ø 25 mm parallel plate with a spacing of 1 mm between the upper and lower plates, an angular frequency of 10 rad/s, and a starting temperature of 58 °C. The complex modulus (G*), phase angle (δ), and rutting factor (G*/(sin δ)) were measured every 6 °C. The high-temperature grade of the asphalts was determined on the basis of (G*/(sin δ)) ≥ 1.0 kPa for the original asphalt and (G*/(sin δ)) ≥ 2.2 kPa for the TFOT residual asphalt.

(2) MSCR test

The DSR was used to apply two different creep stresses, 100 Pa and 3200 Pa, to the asphalt. The samples were loaded at 1 s and unloaded at 9 s for each stress value in each cycle for a total of 10 cycles [37]. Conditions: Ø 25 mm parallel plate with a spacing of 1 mm between the upper and lower plates; the base asphalt and the V-PU modified asphalt were tested at 60 °C.

(3) LAS test

The LAS test was carried out on the base asphalt and the V-PU modified asphalt at 20 °C with Ø 8 mm parallel plate, 2 mm spacing between the upper and lower plates, and a 10 min temperature equilibrium time to obtain the stress and strain of the asphalt.

### 2.6. Fourier Transformation Infrared Spectroscopy (FTIR)

Fourier transformation infrared spectroscopy was carried out on a Nicolet iS10 produced by Thermo Fisher Scientific (Shanghai, China) Co., Ltd. The sample was prepared using the potassium bromide method, and the sample was applied evenly to KBr salt plates and scanned 64 times with a resolution of 4 cm^−1^.

### 2.7. Fluorescence Microscope Dispersion Observation Test

A drop of asphalt sample sandwiched between a slide and a cover slip was heated to 100 °C on a sample heater to give a thin even layer of asphalt sample. The sample was then placed under the object lens, and the distribution behavior of V-PU in the asphalt system was observed using fluorescence microscopy. Micrographs were acquired using a camera.

## 3. Results and Discussion

### 3.1. Rheological Study of V-PU modified Asphalt

#### 3.1.1. High Temperature Grading Performance of V-PU Modified Asphalt

**Figure 4:**
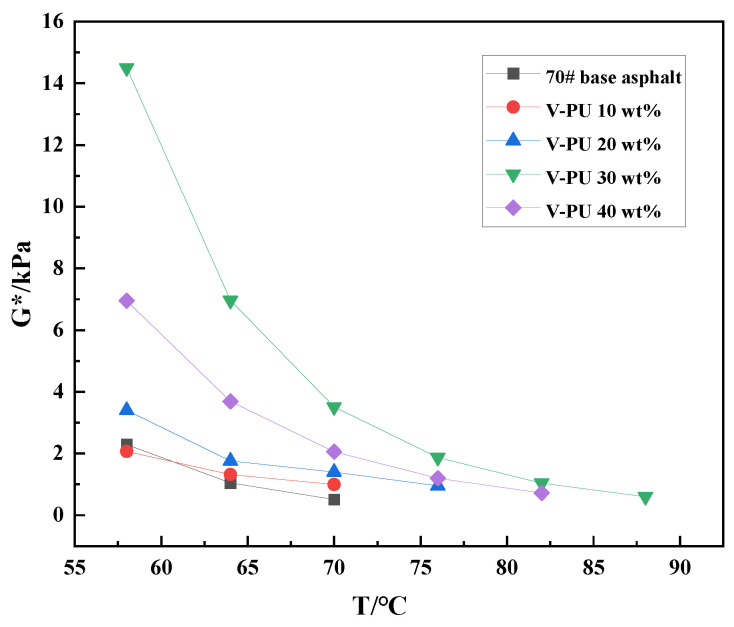
The changes in G* of base asphalt and 10–40 wt% V-PU modified asphalts with temperature.

**Figure 5:**
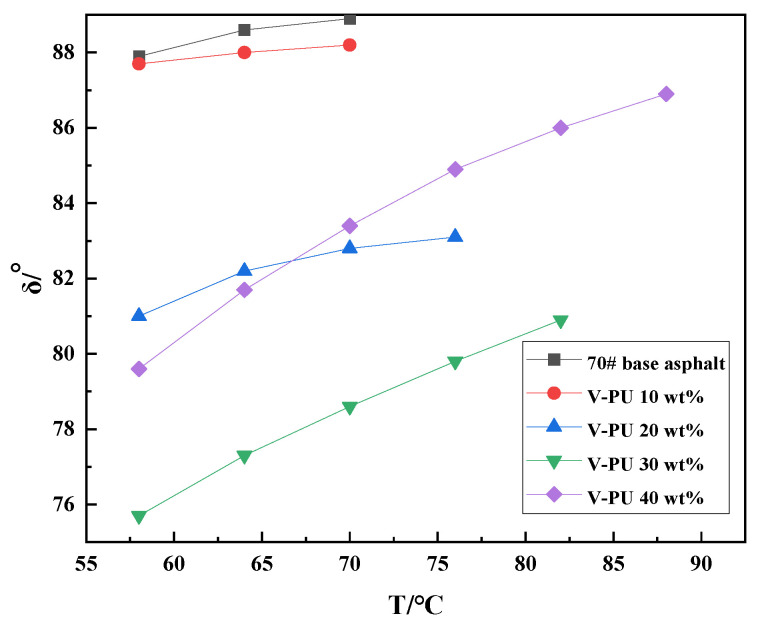
The changes in δ of base asphalt and 10–40 wt% V-PU modified asphalts with temperature.

**Figure 6:**
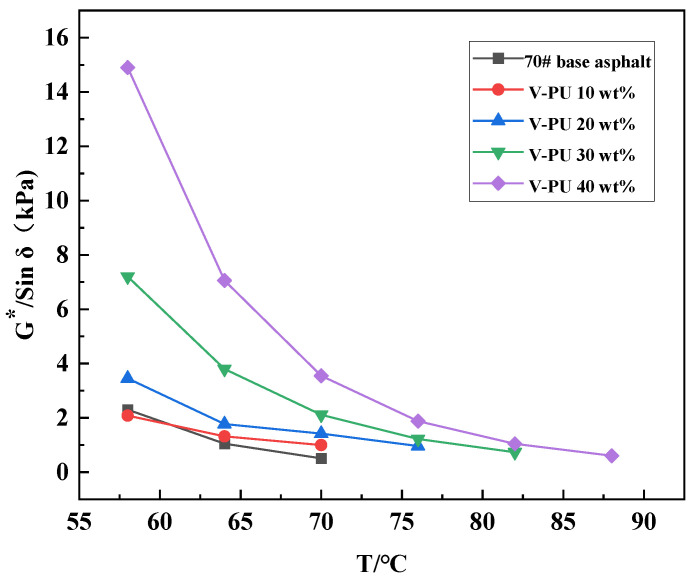
The changes in G*/(Sin δ) of base asphalt and 10–40 wt% V-PU modified asphalts with temperature.

Asphalt is a temperature sensitive material. G* describes the ability of asphalt materials to resist repeated shear stress deformation, and δ is generally used to describe the proportion of unrecoverable viscous components. The changes in G*, δ, and G*/(sin δ) of 70# base asphalt and 10–40 wt% V-PU modified asphalt with temperature are shown in Figure 4, Figure 5 and Figure 6. 

As shown in Figure 4, Figure 5 and Figure 6, the G* of V-PU modified asphalt was much higher than that of the base asphalt. The G* and G*/sin δ of the different asphalts were in the order: 70# base asphalt < 10 wt% < 20 wt% < 40 wt% < 30 wt%; the δ changed in the opposite order, which indicated that the elastic components of the asphalt increased significantly and that the ability to resist deformation was clearly enhanced with the addition of a modifier. This showed the improvement in high and low-temperature performance of the modified asphalt macroscopically. When the content was >30 wt%, the G* and G*/sin δ decreased and the δ increased. This was because the phase transformation dosage was 30 wt% and the modifier was dispersed uniformly in the asphalt when the content was <30 wt%. Asphalt molecules separate V-PU molecules, limiting the contact of the V-PU molecules and providing less opportunity for the V-PU to grow. However, when the content increased to 40 wt%, the content increase allowed the V-PU molecules to come into contact with each other and further reactions and growth to take place. At this point, the system was relatively unstable and two-phase separation occurred, leading to the worst performance. The optimal dosage was considered to be 30 wt%.

High-temperature classification tests were conducted on different original asphalt and TFOT residual asphalt, as shown in Table 4.

As can be observed in Table 4, the high temperature grade of the modified asphalts clearly increased with the increasing V-PU content when the content was below 30 wt%. The high temperature grade of the 30 wt% V-PU modified asphalt was PG 82 ℃, 4 grades higher than that of the base asphalt. The asphaltene content of the asphalt increases, while the content of the resin, aromatic, and saturated components decreases in the long-term high-temperature environment [38]. The irrecoverable viscosity component of the material increased under repeated loading and was more likely to lead to permanent deformation. The V-PU modifier reacted with the base asphalt and formed a three-dimensional network structure; This reconstructs the colloidal structure of the modified asphalt, which is the essential reason for the significant improvement in the high-temperature grade of the modified asphalt. In addition, when the V-PU content exceeded 30 wt%, the base asphalt was the main phase and the V-PU excessively contacted, agglomerated, and settled, resulting in a weakened network structure strength.

#### 3.1.2. Permanent Deformation Resistance of V-PU Modified Asphalt

**Figure 7:**
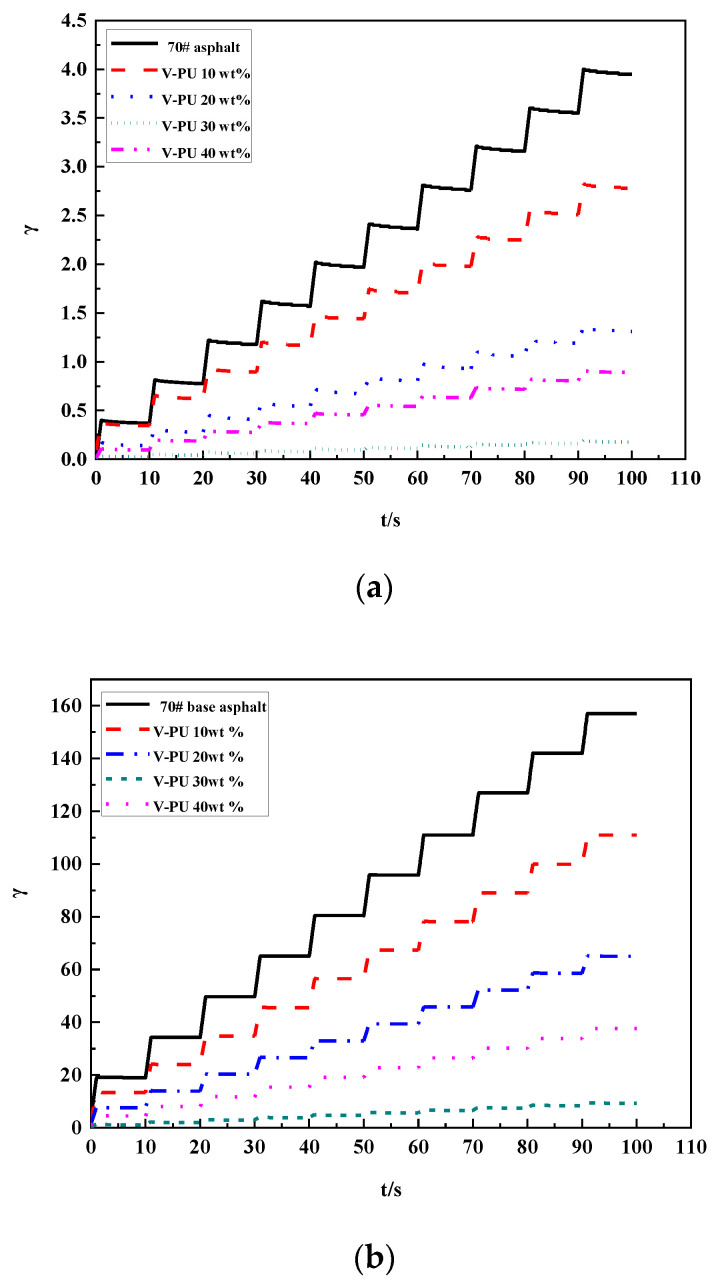
The cumulative creep strain of base asphalt and 10–40 wt% V-PU modified asphalts with the change in load time. (**a**) 60 °C, 100 Pa; (**b**) 60 °C, 3200 Pa.

The MSCR test is currently widely used to evaluate the resistance of asphalt to permanent deformation. Many researchers have demonstrated that the results of the MSCR test and the asphalt mixture rutting test have good correlation [39,40,41,42]. The MSCR experiment was carried out using the DSR at 60 °C, with stresses of 100 Pa and 3200 Pa. The cumulative creep strains of the base asphalt and the modified asphalts with changing load time were obtained, as shown in Figure 7.

As can be seen in Figure 7, the peak strain produced by one load cycle and the residual strain also caused permanent deformation after 10 loading–unloading cycles at the two test stress levels of 100 Pa and 3200 Pa at 60 ℃ in the order: 0 wt% > 10 wt% > 20 wt% > 40 wt% > 30 wt%, indicating that the deformation resistance of V-PU modified asphalt improved and that the 30 wt% V-PU modified asphalt performed best. The MSCR test uses two indexes to evaluate the asphalt resistance to permanent deformation, namely, creep recovery rate (R) and irrecoverable creep compliance (Jnr); the calculation formulas are shown in Equations (3) and (4), respectively. R reflects the elastic recovery ability and deformation resistance of asphalt; Jnr can better characterize the rutting resistance of modified asphalt compared with the rutting factor (G*/sin δ) [43].

(3) $$R_{\tau i=}\frac{r_{\mathrm{pi}}-r_{\mathrm{nri}}}{r_{\mathrm{pi}}-r_{\mathrm{oi}}}\;,$$

(4) $$J_{\mathrm{nr}\tau i}=\frac{r_{\mathrm{nri}}}{\tau }$$

where r_pi_ stands for the peak strain, R_nri_ is the residual strain, and R_0i_ is the initial strain in each loading cycle. τ is creep stress.

**Figure 8:**
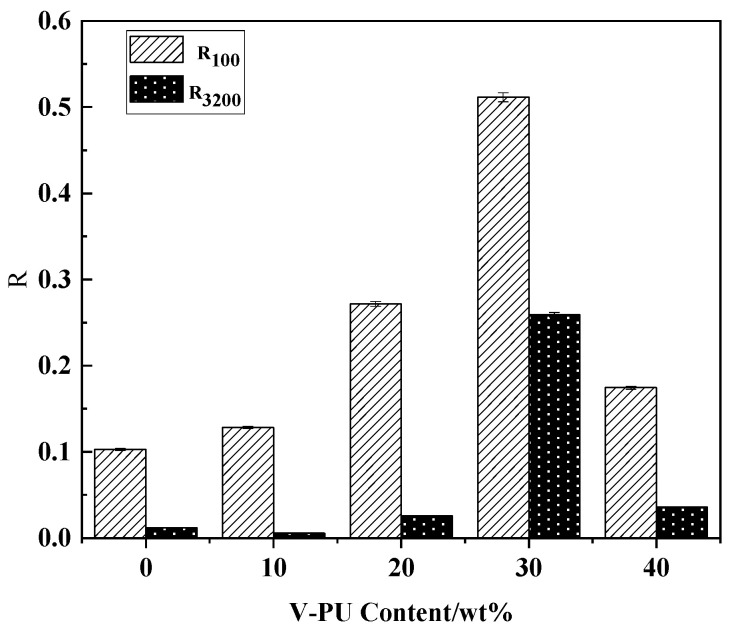
R of base asphalt and modified asphalts with 10–40 wt% V-PU under different stress levels.

**Figure 9:**
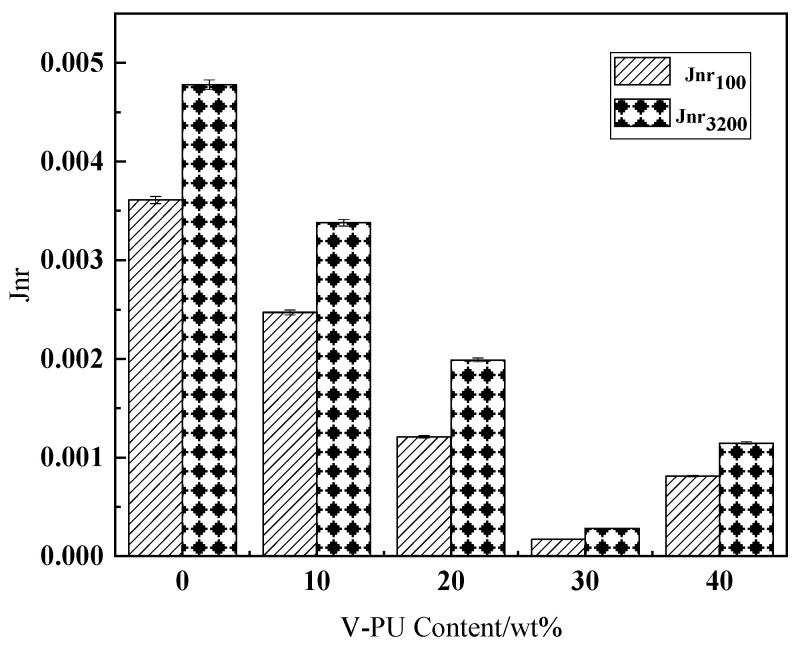
Jnr of base asphalt and modified asphalts with 10–40 wt% V-PU under different stress levels.

The average R and Jnr were calculated under each stress level according to Equations (5) and (6). The calculation results are shown in Figure 8 and Figure 9.

(5) $$R_{\tau }=\frac{\sum_{i=1}^{10}R_{\tau i}}{10}$$

(6) $$J_{\mathrm{nr}\tau }=\frac{\sum_{i=1}^{10}J_{\mathrm{nr}\tau i}}{10}$$

As shown in Figure 8 and Figure 9, the R for the V-PU modified asphalts was significantly higher than that of the base asphalt, while the Jnr was less than that of the base asphalt at the same stress level. This indicated that the V-PU modified asphalt had significantly better resistance to permanent deformation and recovery deformation than the base asphalt. Among the systems tested, the 30 wt% V-PU modified asphalt had the best performance. The addition of V-PU improved the stiffness of the base asphalt, and the increase in R reflected the increase in delayed elasticity after the asphalt modification. The R decreased and the Jnr increased when the stress increased from 100 Pa to 3200 Pa at the same temperature. This was because the greater wheel load produced a deeper rut depth, resulting in permanent deformation. The increasing rate of the Jnr of the base asphalt with stress was slower than that of the modified asphalt, indicating that the modified asphalt was more sensitive to the change in stress. For the V-PU modified asphalt, the change rate of the Jnr of the 30 wt% V-PU modified asphalt was lowest with increasing stress, indicating that the greater the modification effect was, the lower the sensitivity to stress was.

#### 3.1.3. Resistance to Fatigue Cracking of V-PU Modified Asphalt

**Figure 10:**
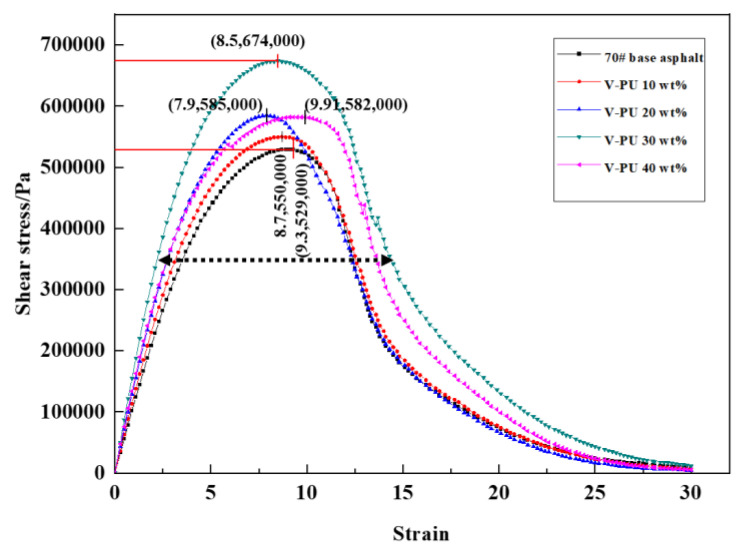
Shear stress–strain of base asphalt and modified asphalts with 10–40 wt% V-PU.

The linear amplitude sweep (LAS) test can be quickly carried out to evaluate the fatigue performance of asphalt materials and the ability of different asphalts to resist load damage. The stress and strain responses of the base asphalt and modified asphalts by an LAS test at a temperature of 20 °C are shown in Figure 10.

The peak of shear stress is defined as the yield stress of the material, and the corresponding strain is the yield strain. Elastic materials can resist deformation before the yield stress but show elastic embrittlement when the yield stress is exceeded. Higher yield stress shows greater resistance to deformation for a certain load. The yield strain corresponding to the yield stress is the deformation degree of the material under the maximum load. The increase in yield strain indicates that the elasticity of the material improves. As can be observed in Figure 10, the yield stress of the different asphalts was in the order: 30 wt% > 40 wt% > 20 wt% > 10 wt% > 0 wt%, and the order of the yield strain was 40 wt% > 30 wt% > 20 wt% > 10 wt% > 0 wt%, which indicates that the addition of V-PU enhanced the fatigue resistance of the asphalt. A certain span of the parabola indicated that the material had a period of yield process. It can be seen from Figure 10 that the order of anti-fatigue deformation performance was 30 wt% > 40 wt% > 20 wt% > 10 wt% > 0 wt%, indicating that the addition of V-PU improved the anti-fatigue deformation of the asphalt, and the effect was most obvious when the content was 30 wt%.

### 3.2. Aging Resistance and Mechanism of V-PU Modified Asphalt

#### 3.2.1. Aging Resistance of V-PU Modified Asphalt

**Figure 11:**
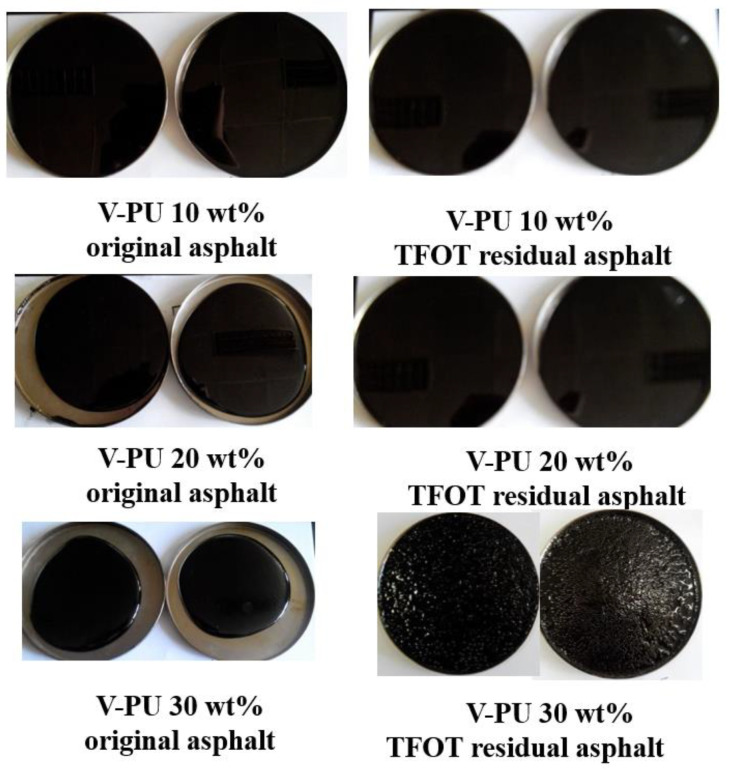
The state of original asphalt and TFOT residual asphalt with different content of V-PU.

Modified asphalt will be oxidized and volatilized during hot processing, storage, transportation, and mixing. It will also undergo complex physical and chemical reactions such as decomposition in contact with the air and on exposure to sunlight and rain during the use of the asphalt pavement. Asphalt aging is an important aspect that affects pavement performance. TFOT at 130 °C was carried out on V-PU modified asphalts, and the results are shown in Table 5. The states of the original asphalts and the TFOT residual asphalts are shown in Figure 11.

As can be seen in Table 5, both the penetration and ductility of V-PU modified asphalts have serious attenuation at 130 °C. As can be observed in Figure 11, the asphalt surface was still smooth and flat, and there was no obvious skinning, coalescence, or particle phenomenon after TFOT at 130 °C when the content of the V-PU was less than 20 wt%. There was obvious particle agglomeration on the surface after TFOT when the content exceeded 30 wt%. The viscosity increased and the fluidity decreased. Studies have shown that gel scaling occurs on the surface of PU modified asphalt above 130 °C [44]. On one hand, castor oil polyol as one of the synthetic monomers contains three ester groups, oxygen functional groups, and unsaturated fat chains with three double bonds, which are prone to oxidation and aging at high temperatures. On the other hand, when the content exceeded 30 wt%, the increase in the content allowed V-PU molecules terminated with –NCO groups to come into contact with each other and react further to grow to make the modified asphalt system unstable and cause two-phase separation. In addition, a high temperature environment for an extended period during aging accelerated this process, leading directly to poor performance after aging.

#### 3.2.2. Aging Mechanism of V-PU Modified Asphalt

**Figure 12:**
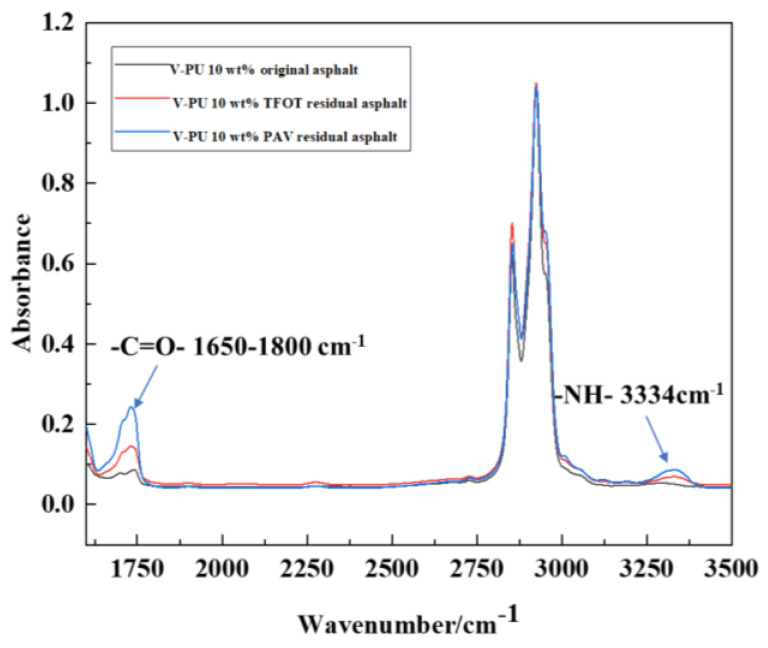
Comparative analysis of FTIR spectrum.

FTIR, gel permeation chromatography (GPC), and fluorescence microscopy dispersion observation analyses were conducted on the original 10% V-PU modified asphalt and the TFOT and PAV residual asphalts. The FTIR spectra are shown in Figure 12.

As shown in Figure 12, the absorption peak at 1650~1800 cm^−1^ of the TFOT and PAV residual asphalts increased, which indicated that the asphalt system produces more carbonyl compounds during the aging process. The absorption peak above 3000 cm^−1^ of the TFOT and PAV residual asphalts was also enhanced. The peak was primarily assigned to the characteristic absorption peak of hydroxyl and carboxyl groups, and the absorption peak at 3334 cm^−1^ was assigned to the stretching vibration peak of –N–H, indicating that hydroxyl and carboxyl compounds were generated during the high-temperature aging; further reactions of excessive –NCO groups occurred to form new compounds during the aging process, which manifested as asphalt sample agglomeration, coalescence, and surface scaling.

The molecular weight distribution of the original 10 wt% V-PU modified asphalt and the TFOT and PAV residual asphalts is shown in Table 6.

Table 6 shows that the molecular weight of the TFOT and PAV residual asphalts, particularly Mz and Mz + 1, was significantly greater than that of the original asphalt, indicating that the functional groups had undergone oxidation and polymerization reactions, generating more complex polymer micelles during the high-temperature aging. Combined with the FTIR test analysis above, this indicates that carbonyl, hydroxyl, and carboxyl oxidizing substances were formed. The polydispersity (d) of the TFOT and PAV residual asphalts also increased, indicating that a variety of complex polymer fragments were formed during the high-temperature aging process.

**Figure 13:**
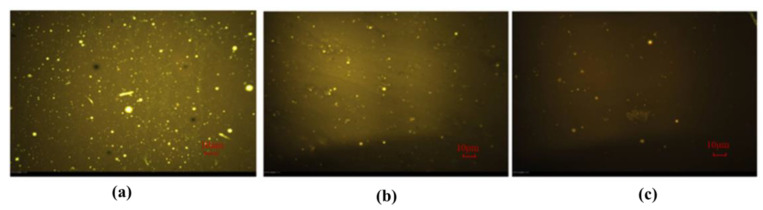
Microstructures of original 10 wt% PU modified asphalt and TFOT and PAV residual asphalts. (**a**) V-PU 10 wt% original asphalt; (**b**) V-PU 10 wt% TFOT residual asphalt; (**c**) V-PU 10 wt% PAV residual asphalt.

The microstructures of the original 10 wt% V-PU modified asphalt and of the TFOT PAV residual asphalts were observed by fluorescence microscopy magnified 100 times, as shown in Figure 13.

Optical microscopy observation was also used as a direct approach to study the distribution behavior of compound particles and the phase interface between the modifiers and the base asphalt at high temperature [19]. Figure 13 shows that the V-PU modifier dispersed uniformly with a good shearing effect and that no obvious phase interface in the original V-PU modified the asphalt system. The particles of the modifier present in the system after the TFOT was tested decreased, and there were large molecular groups such as colloidal agglomerates. Similarly, PAV residual asphalt contained large particle substances, which further indicated that the V-PU in the modified asphalt system experienced re-polymerization, agglomeration, and precipitation during the high-temperature aging.

**Figure 14:**
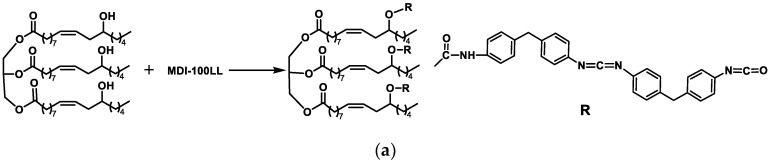

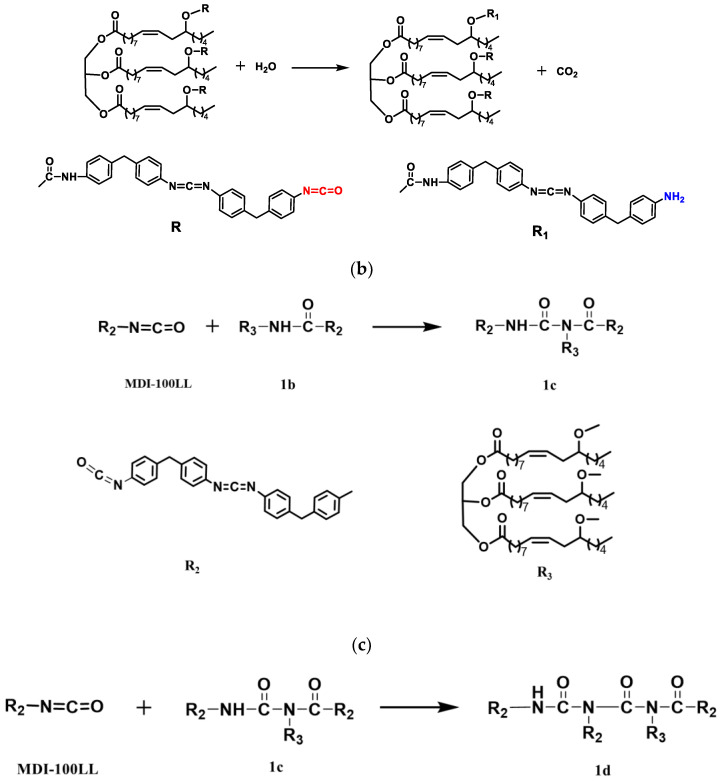
The possible intramolecular gelling reaction and aging process mechanism. (**a**) Prepolymerization of V-PU synthesized from castor oil and MDI-100LL; (**b**) V-PU terminated with -NCO group reacts with water and the active hydrogen group; (**c**) –NHCOO– group in V-PU continues to react with the excessive –NCO group; (**d**) urea formate generated continues to react with excessive NCO group to form biuret.

The V-PU terminated with the –NCO group modified asphalt experienced a complex gelling reaction under the conditions of high temperature, water vapor, and the presence of active hydrogen groups in the asphalt system. It is speculated that carbonyl and carboxyl polymers are generated and a series of reactions such as oxidation and self-polymerization to synthesize urea take place, judging from the FTIR and GPC analysis. V-PU prepolymer can react with water in the air and on the surface of materials and the active hydrogen group in the asphalt system. The –NHCOO– group in the V-PU prepolymer continued to react with the excess –NCO groups to form a urea formate cross-linking bond, and the urea formate generated further reacted with excess –NCO groups to form a biuret at high temperature, finally forming a cross-linked three-dimensional network structure with the asphalt, which improved the performance of the modified asphalt. In addition, the V-PU modifier experienced oxidation, polymerization reactions, chain breakage, and degradation to generate more carbonyl, hydroxyl, and carboxyl compounds and other colloidal agglomerates during the high-temperature aging, resulting in phase separation and aging of the modified asphalt. A possible intramolecular gelling reaction and aging process mechanism are shown in Figure 14.

## 4. Conclusions

V-PU asphalt modifier terminated with –NCO groups was synthesized from renewable castor oil and liquefied MDI-100LL and had good compatibility with the base asphalt. The V-PU was “green” and sustainable, with low cost. The resulting modified asphalt had the advantages of simple, fast preparation with low shearing temperature without several hours of stirring development and has the potential for promotion and application. The following are the key conclusions:

- The high and low temperature performance and fatigue resistance of the modified asphalt improved compared with the base asphalt. The rheological tests showed that increasing the V-PU clearly increased the modulus and the high temperature grade of the modified asphalts, while the phase angle decreased, which indicated that the elastic components of the asphalt increased significantly and that the ability to resist deformation was clearly enhanced.
- The R of the V-PU modified asphalts was significantly higher than that of the base asphalt, while the Jnr was less than that of the base asphalt at the same stress level, which indicated that the V-PU modified asphalt had significantly better resistance to permanent deformation and recovery deformation than the base asphalt.
- The yield stress and yield strain of the V-PU modified asphalts increased, which indicated that the addition of V-PU modifier enhanced the fatigue resistance of the asphalt. The 30 wt% sample showed phase inversion. The 30 wt% V-PU modified asphalt had the best performance.
- Both the penetration and ductility of the V-PU modified asphalt showed marked attenuation after TFOT at 130 °C. Comprehensive FTIR, GPC, and fluorescence microscopy analysis showed oxygenates. The molecular weight, particularly Mz and Mz + 1, significantly increased, and the V-PU molecules agglomerated after the TFOT and PAV tests. V-PU prepolymer can react with water in the air and the active hydrogen in the asphalt system and finally form a cross-linked three-dimensional network structure with the asphalt to improve performance. Oxidation, polymerization, chain breakage, and degradation of the V-PU modifier resulted in phase separation and attenuation of performance.

## Figures and Tables

**Table 1:** Base properties of castor oil.

Properties	Refractivity	Hydroxyl Value (mg KOH/g)	Acid Value (mg KOH/g)	Iodine Value
value	1.478~1.480	164	≤1.0	82~90

**Table 2:** Base properties of liquid MDI-100LL.

Properties	Appearance	Viscosity (25 °C) (mPa·s)	–NCO Content (wt%)	Acidity (wt%)
value	light yellow transparent liquid	25~60	28~30	≤0.04

**Table 3:** Base properties of 70# road petroleum asphalt.

Properties		Technical Requirements	Value
penetration (25 °C, 100 g, 5 s) (0.1 mm)		60~80	65
softening point (5 °C) (°C)		≥45	46.0
ductility (5 cm/min, 15 °C) (cm)		≥100	>100
ductility (5 cm/min, 10 °C) (cm)		≥25	55
dynamic viscosity (60 °C) (Pa·s)		≥160	185
after TFOT	mass loss (%)	≤±0.8	0.075
	penetration ratio (25 °C) (%)	≥61	68
	ductility (5 cm/min, 10 °C) (cm)	≥6	8

**Table 4:** High-temperature classification results of different asphalts.

Asphalt Type	G*/sin δ of Original Asphalt/kPa						G*/sin δ of TFOT Residual Asphalt/kPa						High Temperature Grade/°C
	58 °C	64 °C	70 °C	76 °C	82 °C	88 °C	58 °C	64 °C	70 °C	76 °C	82 °C	88 °C	
70# base asphalt	2.30	1.05	0.510 *	—	—	—	4.32	1.93 Φ	—	—	—	—	PG 58
V-PU 10 wt%	2.08	1.32	0.997 *	—	—	—	5.12	2.33	1.11 Φ	—	—	—	PG 64
V-PU 15 wt%	2.71	1.38	0.744 *	—	—	—	5.50	2.77	1.47 Φ	—	—	—	PG 64
V-PU 20 wt%	3.45	1.77	1.420	0.963 *	—	—	8.83	4.74	2.73	1.68 Φ	—	—	PG 70
V-PU 30 wt%	14.90	7.06	3.550	1.880	1.040	0.605 *	22.30	13.40	8.61	5.84	3.21	2.54	PG 82

* The high temperature grade test requires (G*/(sin δ)) ≥ 1.0 kPa for the original asphalt, otherwise the test must stop. Φ The high temperature grade test requires (G*/(sin δ)) ≥ 2.2 kPa for the TFOT residual asphalt, otherwise the test must stop.

**Table 5:** TFOT results of V-PU modified asphalts with different content of V-PU.

Asphalt Type	Original Asphalt		TFOT Residual Asphalt	
	Penetration (25 °C, 100 g, 5 s) (0.1 mm)	Ductility (5 cm/min, 5 °C) (cm)	Penetration (25 °C, 100 g, 5 s) (0.1 mm)	Ductility (5 cm/min, 5 °C) (cm)
V-PU 10 wt%	117.0	9.8	66.5	2.4
V-PU 15 wt%	113.0	9.4	56.5	3.9
V-PU 20 wt%	85.0	8.3	50.3	3.1
V-PU 30 wt%	63.0	7.7	44.4	2.9

**Table 6:** Molecular weight distribution of original 10 wt% V-PU modified asphalt and TFOT and PAV residual asphalts.

Asphalt Type	Mn	Mw	Mz	Mz + 1	Mv	Polydispersity
original asphalt	871	2453	8451	12,548	2030	2.815700
TFOT residual asphalt	900	2862	9221	17,093	2338	3.177970
PAV residual asphalt	962	2995	9826	17,749	2421	3.447751

## Data Availability

Data are contained within the article.
